# Dacarbazine-encapsulated solid lipid nanoparticles for skin cancer: physical characterization, stability, *in-vivo* activity, histopathology, and immunohistochemistry

**DOI:** 10.3389/fonc.2023.1102269

**Published:** 2023-04-21

**Authors:** Sankha Bhattacharya, Satyam Sharma

**Affiliations:** ^1^ Department of Pharmaceutics, School of Pharmacy & Technology Management, SVKM’S NMIMS Deemed-to-be University, Shirpur, Maharashtra, India; ^2^ Department of Pharmacology and Toxicology, National Institute of Pharmaceutical Education and Research (NIPER), Export Promotion Industrial Park (EPIP), Hajipur, Bihar, India

**Keywords:** solid lipid nanoparticles, ehrlich ascetic carcinoma, melanoma, cancer, wistar rats

## Abstract

**Background:**

This study examined the use of solid lipid nanoparticles (SLNs) to administer Dacarbazine (DTIC) to skin melanoma cells with minimal adverse effects. Melanoma is a tricky skin cancer to cure, and standard chemotherapy has many negative effects. Encapsulating DTIC in SLNs may allow the drug to target melanoma cells without harming healthy cells. The study developed and tested DTIC-loaded SLNs for skin melanoma treatment.

**Methods:**

This study encapsulated Dacarbazine (DTIC) in solid lipid nanoparticles (SLNs). SLNs with reversed micelles were produced utilizing specified ratios of the surfactant Kolliphor^®^ P188 and phosphatidylcholine. To track SLN drug localisation, gold nanoparticles were conjugated to the DTIC. Nanoparticle size and form were examined using DLS and TEM. These approaches ensured SLNs had the correct size and shape for drug delivery.

**Significant findings:**

In the study, various parameters of the developed solid lipid nanoparticles (SLNs) were evaluated, including particle size, zeta potential, polydispersity index (PDI), entrapment efficacy, and cumulative drug permeation. The values for these parameters varied across the different formulations, with particle size ranging from 146 ± 4.71 nm to 715 ± 7.36 nm, zeta potential from -12.45 ± 2.78 mV to -30.78 ± 2.83 mV, PDI from 0.17 ± 0.013 to 0.51 ± 0.023, entrapment efficacy from 37.78 ± 2.78% to 87.45 ± 4.78%, and cumulative drug permeation from 117 ± 4.77 μg/cm^2^ to 275 ± 5.67 μg/cm^2^. To determine the optimal anti-cancer formulation, the DTIC-SLNs-8 nanoparticles were mixed with an optimized concentration of Gellan gum (0.01% w/v) and applied to DMBA-induced skin tumors in rats for six weeks, twice daily. Histopathology demonstrated that DTIC-SLNs-8-treated rats had less keratosis, inflammatory responses, and angiogenesis than free DTIC-treated rats. The development of SLNs may be a promising approach for melanoma treatment due to their improved drug retention over the skin. The optimised anti-cancer formulation DTIC-SLNs-8 showed improved efficacy with minimal side effects as compared to free DTIC.

## 1 Introduction

Dacarbazine is a chemotherapy drug that is used to treat metastatic malignant melanoma (1–4), a type of skin cancer. The drug works by alkylating and arresting the DNA of cancerous cells, disrupting their normal function. Dacarbazine is a 1H-imidazole-4-carboxamide molecule that has been modified with a 3,3-dimethyltriaz-1-en-1-yl group (5, 6). It can also be used to treat soft-tissue sarcoma (7, 8). However, its effectiveness in treating malignant melanoma is limited due to the hydrophilic and acidic nature of cancerous tissue, which requires higher doses of the drug to be administered and can lead to drug resistance and non-selective cell toxicity (9, 10).To attain maximum benefits with minimum side effects, a nanotechnological approach to delivering drug precisely in the site of action is desired (11, 12). Within this decade, many nanoparticular approaches has been investigated to deliver drug more effectively into the skin cancer target sites. As per Soliman et al. (2018), Fluorouracil-Loaded Gold Nanoparticles were prepared to treat skin cancer (13). The in-vivo anticancer activity studies were performed against A431 tumor-bearing mice; Cancer Xenograft Model. As per Rafaela Malta et al. (2021) 1-carbaldehyde-3,4-dimethoxyxanthone (LEM2) lipid nanoparticles can be used for the treatment of melanoma (14). This nanoparticle had good anticancer effects against melanoma A375 cell lines. To improve aqueous solubility and poor bioavailability of LEM2, the Malta et al, encapsulate LEM2 in the form of lipid nanoparticles by homogenization (HPH) and ultrasonication method. The anticancer activity of the prepared formulation was tested against melanoma A375 cell line, which indicates the application of lipid nanoparticles as a carrier to LEM2 enhances the efficacy of the drug. As per Patel Gayatri et al. (2020), by ionic gelation method and in the presence of chitosan and sodium tripolyphosphate (TPP), 5-Fluorouracil loaded nanoparticles were prepared. The cytotoxicity studies were carried out by MTT assay using A375 basal cell carcinoma cell-line (15). For topical drug delivery, the solid lipid nanoparticles approach would be the best possible solution to treat metastatic malignant melanoma (16, 17).The prepared solid lipid nanoparticles can be stabilized by using suitable surfactant and co-surfactants. Due to the lipophilic nature of the solid lipid nanoparticles (SLNs), the SLNs can encapsulate hydrophobic drugs (18). However, SLNs approach in many cases counterfeits for hydrophilic chemotherapeutic drugs, I.e., Epothilone, Fluorouracil, Gemcitabine, Hydroxyurea. Few efforts have been made to date using dacarbazine to eradicate melanoma (DTIC). According to Qin Shen et al. (2008) (19), gold nanoparticles were created using DNA bases and dacarbazine to enhance the performance of biosensors. These biocompatible nanoparticles have been shown to be effective in the treatment of cancer. Dacarbazine-Loaded AuSiO2 Nanoparticles have a significant impact on the treatment of melanoma, according to Arman Esmail Zadeh et al. (2022) (20). They used flow cytometry and 3-(4,5-Dimethylthiazol-2-yl)-2,5-diphenyltetrazolium bromide (MTT) assays in their research, and they came to the conclusion that DTIC@AuSiO2 nanoparticle treatment significantly increased the induction of apoptosis in B16F10 melanoma cells.

Mahdi Barjasteh et al. (2022) (21) claim that Dacarbazine (DTIC) can be contained within the MIL-100(Fe) metal-organic framework (MOF). Such controlled-release nanoparticles would be a successful melanoma therapy. It was coated with polyethylene glycol (PEG) to make it more cytotoxic, and then the nanoparticles were tested for cytotoxicity against A375 cell lines. PEG can disrupt the cell membrane and cause leakage of intracellular contents, leading to cell death. Additionally, PEG has been shown to induce the production of reactive oxygen species (ROS) in cancer cells, which can also cause cytotoxicity.

Various approaches have been proposed to overcome the above-mentioned problems, including encapsulation of the drug into liposomes (22), polymers (23, 24), micelles (25–27) etc. In addition to these vectors for the delivery of drugs, core-shell SLNs or Stable-Projectile SLNs were also independently proposed as carriers to increase Dacarbazine’s drug delivery profile and anticancer effects.

In normal solid lipid nanoparticles (SLNs), lipids can be prepared in colloidal form in the 50-1000 nm range, but in core-shell SLNs, nanoparticles develop with hydrophilic shells loaded with anhydrous micelles surrounding a core comprising of loaded drug (28–31). As a result, in core-shell SLNs, overturn micelles and a core-shell composed of drug loading may be possible, allowing for a dual dose of drug release, one for initial burst effect and the other for sustained release. These phenomena can resemble bullets or rockets when it comes to projecting a target. As a result, renaming core-shell SLNs to projectile SLNs might be a good way to justify formulation characteristic nature of the modified SLNs.

In this study, a novel approach was taken by encapsulating Dacarbazine within stable solid lipid nanoparticles (SLNs) for the first time. The encapsulation resulted in an increase in skin permeation. The optimized Dacarbazine-encapsulated stable SLNs demonstrated the ability to deliver drugs in a limited time frame. An in-depth investigation was conducted to evaluate the encapsulation efficiency, particle size, zeta potential, and stability of the prepared SLNs. The anticancer activity of the SLNs was also evaluated using a DMBA-induced tumor model in the skin. However, further research is needed to understand the effects of the SLNs on immune cell infiltration and induction of apoptosis in tumor sections. A xenograft mouse model will also be developed to conduct additional studies.

## 2 Materials and methods

### 2.1 Materials

Dacarbazine was obtained from Mumbai-based Neon Laboratories Limited. BASF Chemicals, Bengaluru, India, supplied Kolliphor^®^ P188. Sigma-Aldrich, Bengaluru, India, supplied glycerol monooleate, phosphatidylcholine, ethyl alcohol, and polysarcosine. Sisco Research Laboratories Pvt. Ltd., Mumbai, supplied the gellan gum, chitosan, and phosphate buffer tablets. Sigma-Aldrich, Bengaluru, India, supplied the Gold (III) chloride trihydrate (HAuCl4.3H2O). Rest all or most of the chemicals and reagents of specific analytical grades that have been used.

### 2.2 Methods

#### 2.2.1 Preparation of dacarbazine encapsulated shell-enriched solid lipid nanoparticles

After necessary modification of the procedure described in Rasha A. Khallaf et al. (2016), the SLNs were formulated (32). The required quantity of Dacarbazine(15mg), glycerol monooleate, phosphatidylcholine was added into ethyl alcohol (15ml, 70%v/v) to form the organic phase. To form, aqueous phase, varying amount of Kolliphor^®^ P188 (%w/v) added into 75mL of warm dextrose 5% water (**Table 1**). Kolliphor^®^ P 188 can be used in the nanoemulsion to enhance the emulsification process and increase the solubility of DTIC. Additionally, Kolliphor^®^ P 188 may raise the contact angle of the dispersed particles, aiding in particle dispersion and enhancing wettability. Kolliphor^®^ P 188 could ultimately increase the bioavailability of a drug that isn’t very water soluble (33).

**Table 1:** Components of DTIC-SLNs with resultant encapsulation efficiency of different Dacarbazine solid lipid nanoparticles (means ± S.D, n=3).

Formula number	Dacarbazine (mg)	Glycerol monooleate (mg)	Phosphatidylcholine (mg)	Kolliphor® P188 (%w/v)	EE (%)
**DTIC-SLNs-1**	15	75	5.5	1.0	51.56±1.78
**DTIC-SLNs-2**	15	75	5.5	1.2	58.35±3.56
**DTIC-SLNs-3**	15	75	5.5	1.4	64.67±4.67
**DTIC-SLNs-4**	15	75	7.5	1.0	56.78±3.78
**DTIC-SLNs-5**	15	75	7.5	1.2	66.78±4.78
**DTIC-SLNs-6**	15	75	7.5	1.4	67.56±2.67
**DTIC-SLNs-7**	15	75	10	1.0	70.45±4.78
**DTIC-SLNs-8**	15	75	10	1.2	81.67±2.78
**DTIC-SLNs-9**	15	75	10	1.4	87.45±4.78
**DTIC-SLNs-10**	15	75	10	–	37.78±2.78
**DTIC-SLNs-11**	15	75	–	1.4	43.78±2.67

Prepared organic phase added dropwise into the aqueous phase and homogenized at 13,500 RPM for 25 min using T 25 digital ULTRA-TURRAX^®^ Homogenizer (IKA, Germany). The resultant white suspension was instantly transferred into 25 mL ice-cooled distilled water and stirred using REMI 5 MLH Plus Magnetic Stirrer for 2h at 1000 rpm, maintaining the temperature at 2-5°C. Total 11 formulations prepared (DTIC-SLNs-1 to DTIC-SLNs-11), out of which two formulations was kept as control, i.e., One without phosphatidylcholine and another without Kolliphor^®^ P188 (**Table 1**). The resultant DTIC-SLNs were washed thice using a refrigerated centrifuge (REMI, India) at 20000 rpm. After washing, prepared pallets were dispersed in 50mL of 2.5% w/v d-trehalose solution and kept at -85°C for two days before lyophilization (Esquire Biotech Lyophiliser, India) at -40°C. After lyophilization, the resultant free-flowing powder was stored at 4°C for further use.

#### 2.2.2 Preparing DTIC-capped gold nanoparticles

The gold nanoparticles (100 nm) were purchased from Sigma-Aldrich, Bengaluru, India. As a functionalized or conjugation kit, these gold nanoparticles serve as a tracer material in this experiment. The experiment was performed after necessary modification of the preparative method described in Mohamed et al., 2012 (34). The 0.01 M of Dacarbazine (75mg) was added into 75mL of gold nanoparticles solution, which was previously synthesized using citrate reduction of HAuCl_4_.3H_2_O, as described in Mohamed et al., 2012. To enhance the contact between Dacarbazine and synthesized gold nanoparticles, ultrasonication was performed for nanoparticle solution for 15 minutes. Furthermore, this nanogold encapsulated solution (75µL, 0.2mM) was dispersed into methanol in the presence of required quantities of glycerol monooleate and phosphatidylcholine and process as per the preparative procedure described in Dacarbazine encapsulated shell-enriched solid lipid nanoparticles.

### 2.3 Encapsulation efficiency of the SLN

The lyophilized Dacarbazine encapsulated shell-enriched solid lipid nanoparticles (40mg) were dissolved in 20mL of 75% v/v methanol, and the temperature was maintained up to 65°C in a water bath for 45min. The lipid started leached out when the temperature was reduced to 20-25°C. Furthermore, the obtained mixture was centrifuged at 6000 rpm. The obtained supernatant was diluted in distilled water and spectroscopically by LAMBDA XLS+Spectrometer (PerkinElmer Fremont, CA, USA) at 328 nm. As per the method described in Mihaela chişa, et al., 2016 (35), entrapment efficiency was performed. The entrapment efficacy of the prepared formulation was calculated by following equations:


Encapsulation efficiency(%)=(Mass of DTIC encapsulated in nanoparticles/total mass of nanoparticles)×100

### 2.4 Particle size and zeta potential

Delsa Nano C instruments (Beckman Coulter, USA) measure the particle size, polydispersity index (PDI) and zeta potential of the prepared DTIC-SLNs formulations. The emulsion of the nanoparticles was diluted with 0.57 mL of double distilled water and vortexed for 4 minutes to prevent bubbling. The particle size, PDI were measured using Dynamic Light Scattering (DLS) and Zeta potential was measured by Electrophoretic Light Scattering (ELS) at 25°C.

### 2.5 Transmission electron microscopy

TEM electron microscopy (TEM) was used to determine the morphology of the DTIC-SLNs formulations and obtain high-quality images. The TEM instrument used was a Hitachi 7500 from Japan. Prior to TEM analysis, the nanoparticles were coated with carbon and placed on a stained copper grid coated with phosphotungstic acid (1%). The images obtained from TEM studies were analyzed using digital micrograph-generated software.

### 2.6 Diffusion study of the SLN both in PBS and in different gel bases

Using a modified Franz cell system (36), the permeation studies of DTIC-SLNs formulations was investigated. In the upper donner compartment, 0.2µm cellulose nitrate membrane were placed, which eventually sperate the donor compartment and receptor compartment. The 10mg DTIC-SLNs were suspended into a 2mL phosphate buffer solution and placed into the donor compartment. The receptor compartment was filed with 20mL of pH 7.4 phosphate buffer solution. While stirring at 100rpm, a 0.5mL sample was extracted from the receptor compartment at a specific time interval and filtered. The filtrated was measured spectroscopically (PerkinElmer Fremont, CA, USA) at 328 nm. The cumulative amount of the drug permeated per unit area of the nitrate membrane after 24h (Q24 in μg/cm2), the permeation initiation time (lag time in min), and permeability coefficient (Kp) were calculated. The effects of gel base on permeability can be measured by incorporating 25mg of DTIC-SLNs-9 formulation into multiple cationic gel bases, i.e., Gellan gum (0.01%w/v), Chitosan (0.5% w/v), Polysarcosine (2.5% w/v), respectively. Prior to use, the prepared gels were left to equilibrate at 0-4°C for 24h.

### 2.7 Stability study

Stability studies were conducted as per International Council for Harmonisation of Technical Requirements for Pharmaceuticals for Human Use (ICH). Pharmaceutical development: Q8(R2) current step 4 version. Geneva: ICH; 2009.guidelines (37). Measuring UV-VIS spectroscopic encapsulation efficacy (PerkinElmer Fremont, CA, USA) at 328 nm and investigating zeta-potential and particle size of optimized DTIC-SLNs-8 utilizing Delsa Nano C instruments (Beckman Coulter, USA) for 9-months, the stability studies were performed.

### 2.8 *In-vivo* studies

#### 2.8.1 Induction of tumor by DMBA and SLN treatment

An experiment was conducted on rats to study the effects of topical application of 7,12-Dimethylbenz[a]anthacene (DMBA) and Terephthalic acid (TPA) on skin carcinogenesis. The dorsal skin of the rats, measuring 3 x 3 cm in area, was shaved using an electrical clipper and divided into thee groups, each consisting of 10 rats. A fresh DMBA solution was prepared by dissolving 250 µg in 500 µl of acetone. Group I received a vehicle + DMBA solution, while Groups II and III received a single topical application of DMBA. After two weeks of DMBA application, TPA (62.5 µg in 500 µl of acetone) was applied topically thee times per week for 16 weeks to endorse skin carcinogenesis. Group II then received Dacarbazine DTIC-SLNs-8 with Gellan gum (0.01%w/v) twice daily, and Group III received a twice-daily dose of free DTIC with Gellan gum (0.01%w/v). The tumor volume and body weight of all rats were monitored weekly thoughout the experiment. The tumor volume was determined using Vernier caliper, and at the end of 18 weeks, all rats were sacrificed. Tumor tissues were collected, and half were fixed in 10% formalin, while the remaining tissues were processed for further biochemical analysis. The study was conducted in accordance with the Helsinki protocol, Committee for the Purpose of Control and Supervision of Experiments on Animals (CPCSEA) norms, institutional regulations, and national criteria for animal experiments, and was approved by the Institutional Animal Ethics Committee (1410/c/11/CPCSEA) of Deshpande Laboratories in Bhopal, India.

### 2.9 Immunohistochemical analysis

The 10% formalin-fixed tumor tissues were embedded in paraffin and sectioned at 4 µm. The slides were deparaffinized and rehydrated with ethanol. Next, the slides were incubated with anti-Ki-67 primary antibody. Followed by HP/streptavidin conjugated secondary antibody, DAB substrate were added to the slides and counterstained with hematoxylin. The slides were then observed under optical microscope. To calculate proliferative indices, 15 randomly selected microscopic (40× objective) fields in each group were calculated by the total number of cells divided by the number of Ki-67- positive cells.

### 2.10 Statistical analysis of IHC

Statistical Analysis All data are expressed ad mean ± standard deviation (SD). Significant differences were assessed using Student’s t-test and p value < 0.05 was considered as statistically significant.

### 2.11 Histological assessment of skin melanoma

#### 2.11.1 Hematoxylin and eosin staining

Skin areas of DMBA induced skin tumors were fixed in formalin and embedded in paraffin. The excised tumour tissue was fixed in 15% formaldehyde solution. Prior to preparing routine paraffin-block for cancer tissues in Semi-Automatic Rotary Microtome Model RMT-35 model, the tumour tissues were dehydrated. The tissues were dissected up to 4µm and further stained with hematoxylin and further eosin was used as counterstaining agent. Parameters such as tissue necrosis, hemorrhagic areas, reaction associated to inflammation and hyperkeratosis were observed by an independent observer. First, dewaxing was performed in xylene. Skin tissues were sequentially rehydrated with graded ethanol and tap water. Harris Hematoxylin and Eosin (HE) was then used to stain the skin tissues, followed by a wash in tap water for 30min. The skin tissues were then dehydrated with incubations ethanol and xylene. The same staining procedure were repeated and pathological investigations were done in radical biological microscope (Model: RXLr-5 + RFM-4), India.

### 2.12 Statistical analysis

The collected data were statistically evaluated using a t-test for unpaired data; p<0.05 was considered significant.

## 3 Results and discussion

### 3.1 Encapsulation efficiency

#### 3.1.1 Effect of Kolliphor^®^ P188 and phosphatidylcholine on encapsulation efficiency

**Figure 1:**
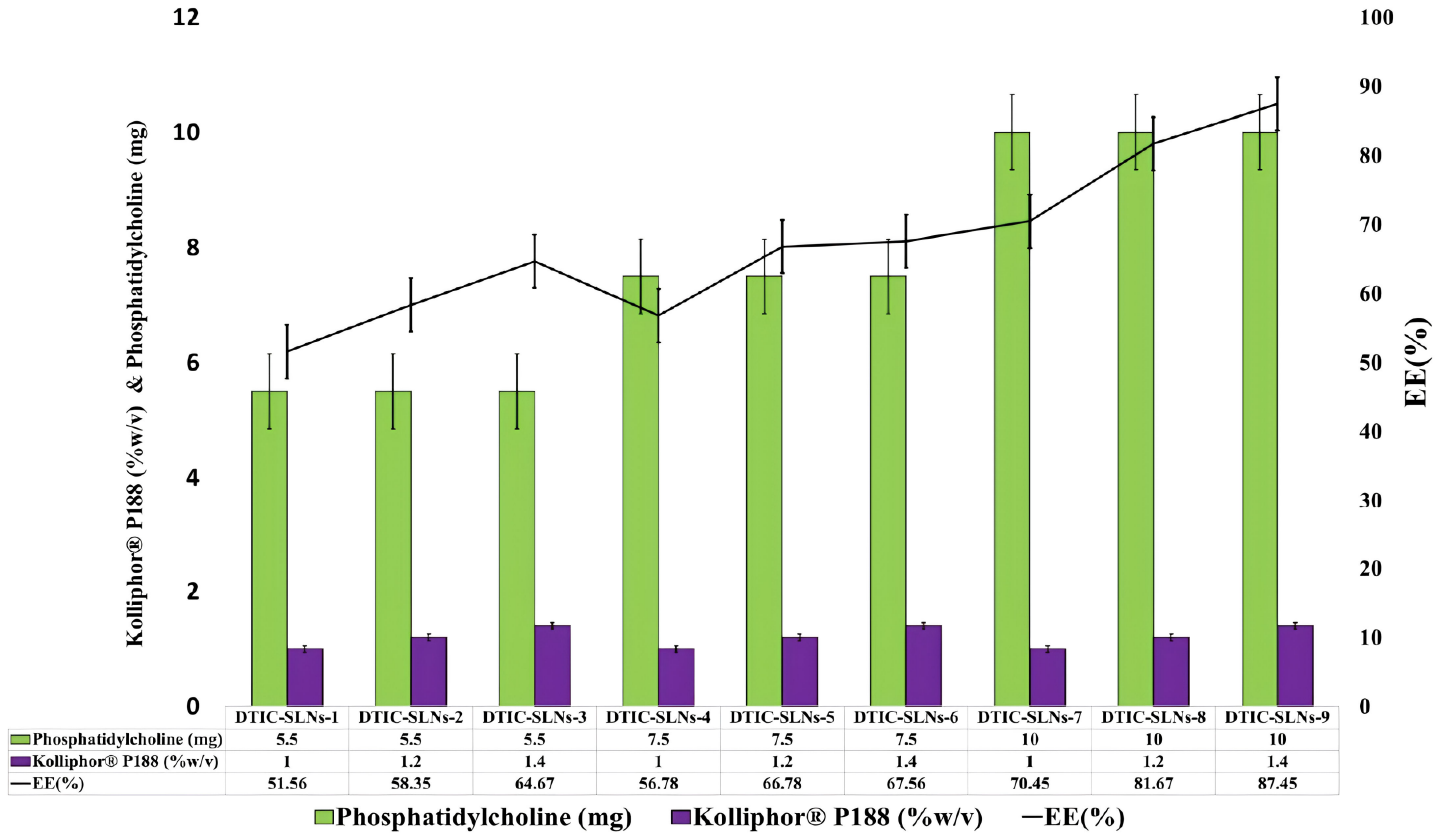
Phosphatidylcholine (mg) and Kolliphor^®^ P188 (w/v%) impact on encapsulation efficacy percentage were correlated.

According to encapsulation efficiency equation, the encapsulation efficiency inside the DTIC-SLNs was determined as a ratio between the volume of drug detected in the DTIC-SLNs and the initial amount of DTIC used. **Table 1** emphasized the percentage of DTIC encapsulated within DTIC-SLNs-1 to DTIC-SLNs-11. At the optimum amount of Phosphatidylcholine (10mg) and Kolliphor^®^ P188 concentration (1.4%) the maximum encapsulation efficacy achieved (87.45 ± 4.78%). In the absence of phosphatidylcholine and Kolliphor^®^ P188, the encapsulation efficacy (%) was found to be marginal, i.e., in the absence of Kolliphor^®^ P188 in DTIC-SLNs-10, the encapsulation efficacy (%) was found to be 37.78 ± 2.78% while in the absence of phosphatidylcholine, DTIC-SLNs-11 efficacy was 43.78 ± 2.67%.The effects of two independent variables, phosphatidylcholine (mg) and Kolliphor^®^ P188 concentration (w/v%), on the encapsulation efficacy percentage of DTIC-SLNs-1 to DTIC-SLNs-11 formulations, were examined. According to **Figure 1**, increasing the concentration of Kolliphor^®^ P188 (% w/v) from 1.0% to 1.4% will improve the encapsulation efficacy percentage of DTIC-SLNs-1 (51.56 ± 1.78%) to DTIC-SLNs-9 (87.45 ± 4.78%) (p<0.05). The outcomes of **Figure 1** clearly indicate that increasing the concentration of Kolliphor^®^ P188 (%w/v) could improve encapsulation efficacy in the presence of phosphatidylcholine. However, in the absence of phosphatidylcholine (DTIC-SLNs-11), struggled to manage the EE % of DTIC. Contrariwise, increase amount of phosphatidylcholine in the presence of Kolliphor^®^ P188, increases DTIC encapsulation efficacy percentage. According to unpaired t-test results, increasing the amount of phosphatidylcholine from 5.5 mg to 10 mg has little effect on encapsulation efficacy (p>0.05) percentage.

As per Mangesh R. Bhalekar et al. (2017) for the treatment of rheumatoid arthitis, solid lipid nanoparticles containing piperine was prepared by melt emulsification method. The macrophagic TNF α secretion was found to be significantly decreased as compared to arthitic control group; indicating accumulation of piperine SLNs in the inflamed site. The results were well explained by Bonferroni multiple comparison test and unpaired t- test was used when compared test 1 and test 2 (p<0.0001) (38). Antonio Leonardi et al. (2014) investigated the effect of different surfactants on lipid nanoparticle ocular tolerability. The nanocarrier created with Kolliphor^®^ P188 was found to be more venerable and safer. Up to the highest tested surfactant concentration (0.4%, w/v), such nanoparticles have no significant irritation effects on the ocular surface (39).

As a result, Kolliphor^®^ P188 can be considered to have a safer profile when it comes to nanocarrier preparation.

The findings of the current research clearly indicate that both Kolliphor^®^ P188 and phosphatidylcholine play a significant role in entrapment efficacy, although Kolliphor^®^ P188 plays the most important role in increasing entrapment efficacy To enhance the encapsulation efficacy of DTIC loaded solid lipid nanoparticles, phosphatidylcholine was used as a co-surfactant. To improve stability of the nanoparticles Kolliphor^®^ P188 can be act as a stabilizer. Because of its amphiphilic nature, phosphatidylcholine forms reverse dehydrated micelles in the organic phase, however when added to the aqueous phase, it inverts and escapes from DTIC-SLNs. As a result, a stabilizer cum surfactant like Kolliphor^®^ P188 could play a critical role by scaffolding micelles inside the shell area of the DTIC-SLNs, preventing phosphatidylcholine from escaping into the aqueous region and increasing nanoparticle stability. Furthermore, the use of Kolliphor^®^ P188 increases the system’s colloidal stability.

Kolliphor^®^ P188 has been shown to act as a surfactant and a stabilizer in emulsion systems. It reduces the interfacial tension between the oil and water phases, which leads to the formation of small droplets and enhances the stability of the emulsion system. Additionally, it has been demonstrated that Kolliphor^®^ P188 can also stabilize lipid-based nanoparticle systems. Furthermore, in many studies the researchers investigated the effect of Kolliphor^®^ P188 on the stability of a protein formulation. Researchers found that the addition of Kolliphor^®^ P188 increased the colloidal stability of the formulation, as demonstrated by a decrease in particle aggregation and an increase in particle size uniformity. In one of the recnt study investigated the effect of Kolliphor^®^ P188 on the stability of a liposomal system. The researches found that the addition of Kolliphor^®^ P188 improved the colloidal stability of the liposomes by reducing particle aggregation and maintaining a uniform particle size distribution.

### 3.2 Particle size and zeta potential measurement

Riddick, 1968 first defined the theshold zeta potential value of agglomeration within the dispersion, which is in between -20 mV to -11mV. As per Sobia Razzaq et al. (2021), paclitaxel multi-functional papain anchored polymeric amphiphilic micelles were papered. Which had negative zeta potential (−29 ± 3.85mV) with higher encapsulation efficacy (80 ± 3.29%) (40). Putthiporn Khongkaew et al. (2021) combined propolis wax with surfactants such as Brij 721, Cremophor WO 7, Myrj 52, Kolliphor^®^ P188, and Tween 80 to develop Solid Lipid Nanoparticles (SLN). Brij 721 and Myri 52, when used at a 20% concentration, can be used to make SLN and have good vitamin E preservation properties (size: 451.2 nm and 416.8 nm, zeta potential: - 24.0mV and - 32.7mV, respectively; percent EE: 92.32% and 92.00%) (41).

**Figure 2:**
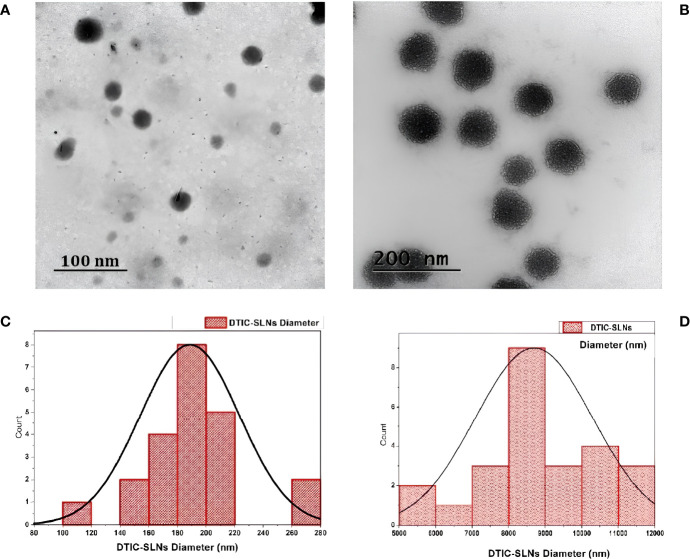
Transmission electron microscopy (TEM) images of the prepared DTIC-SLNs-9 at 100 nm scale **(A)** and 200 nm scale **(B)**. The histogram of DTIC-SLNs diameters indicating 188.70nm as average diameter with 34.66nm standard deviation **(C)**. The mean surface area of DTIC-SLNs was found to be 8693.63 nm^3^ with a standard deviation of 1600.66 nm^3^ **(D)**.

In the current experiment, except DTIC-SLNs-8 to DTIC-SLNs-11, rest all the formulations zeta potential are relatively low and within the Riddick theshold (-19.56 ± 2.82mV to -12.45 ± 2.78 mV). Due to the shielding effects of nanoparticles owing to the hydrophilic corona of Kolliphor^®^ P188, the zeta potential values of nanoparticles are inadequate to produce electrostatic stabilization. However, from the transmission electron microscopy, it is clearly indicating that, Kolliphor^®^ P188 offers additional steric and electrostatic stabilization within formulae. The presence of Kolliphor^®^ P188 increases steric stabilization within the surface of the nanoparticles, even though the particles have an inadequate charge to generate electrostatic stabilization. Particle size analysis by though Dynamic Light Scattering (DLS) system indicating that (**Table 2**), almost all the formulation had smaller particle size ranging from 146 ± 4.71 nm to 415 ± 10.89 nm and PDI from 0.17 ± 0.013 to 0.28 ± 0.012. However, formulation DTIC-SLNs-10 & DTIC-SLNs-11 showing aggravated zeta potential, particle size and polydispersity index, which is due to the absence of Kolliphor^®^ P188 and phosphatidylcholine within one of each formulation. In comparison to the average mean diameter and surface area of the collected morphological images from TEM analysis (**Figures 2A–D**), the average mean diameter obtained by the Dynamic Light Scattering (DLS) process was slightly higher. Reversed micelles are water droplets with a diameter of 1-10nm that form in the presence of a suitable surfactant. The surfactant molecules exposed the organic phase by rearranging polar parts to the inner side and non-polar regions. The nature of the surfactant used during the preparation of reversed micelles, according to Mehta, Ganguli et al. (2021), will influence the shape of the formulation. Hydrophobic alkyl chains and cationic, anionic, and non-ionic hydrophilic head groups were used to create reversed micelles in this experiment. Spherical shell micelles were studied using Small Angle X-ray Scattering (SAXS) (42).

**Table 2:** Zeta potential, Size and polydispersity index values of different SLN formulas (mean ± SD, n=3).

Formula number	Zeta potential (mV)	Size (nm)	Polydispersity index
**DTIC-SLNs-1**	-12.45±2.78	415±10.89	0.23±0.012
**DTIC-SLNs-2**	-13.78±1.78	359±7.78	0.25±0.017
**DTIC-SLNs-3**	-14.25±3.89	205±6.78	0.28±0.012
**DTIC-SLNs-4**	-15.67±3.89	376±8.72	0.27±0.010
**DTIC-SLNs-5**	-19.56±2.82	154±3.89	0.24±0.014
**DTIC-SLNs-6**	-17.67±2.89	193±7.22	0.26±0.016
**DTIC-SLNs-7**	-16.78±1.89	258±5.81	0.27±0.018
**DTIC-SLNs-8**	-23.67±3.98	146±4.71	0.17±0.013
**DTIC-SLNs-9**	-25.56±2.78	198±4.35	0.24±0.112
**DTIC-SLNs-10**	-30.78±2.83	821±6.1	0.51±0.023
**DTIC-SLNs-11**	-21.67±3.67	715±7.36	0.27±0.001

**Figure 3:**
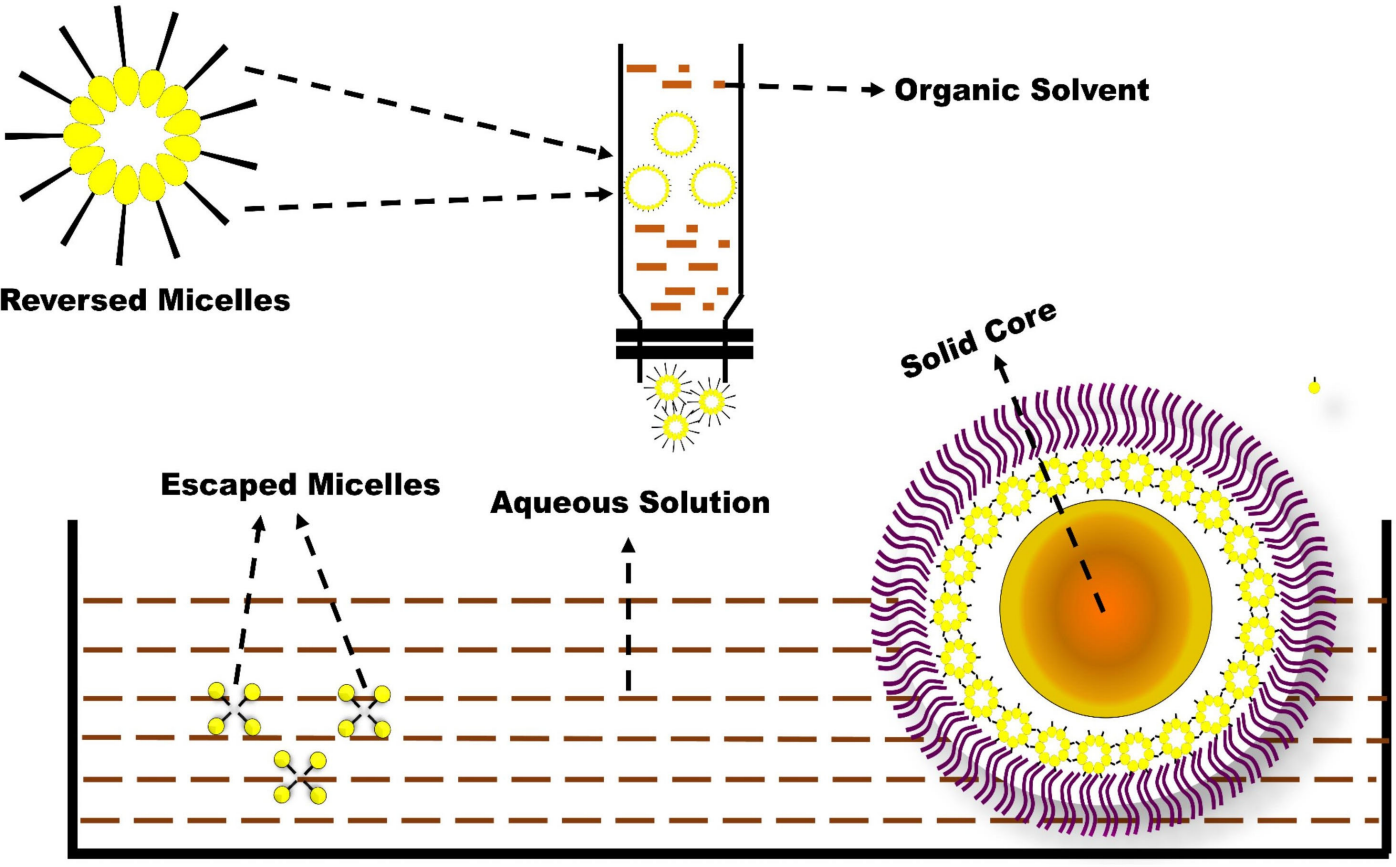
Mechanism and Formation of Micelles while formulating DTIC-SLNs.

In the current experiment, due to the steric hindrance, the aggregation tendency of the nanoparticles was less prominent. The use of Small Angle X-ray Scattering (SAXS) to examine the morphological behaviour of reversed micelles was not considered in this study. However, using transmission electron microscopes (TEM) the micelle formation can be theorized and examined. From the TEM image (**Figure 2D**) It was assumed that nanoparticles had developed two distinguished layers, with the outer hydrophilic layer representing the shells and the inner hydrophilic layer representing the core, composed of loaded Dacarbazine (DTIC). Dacarbazine (DTIC) is believed to be present in these layers or shells (43). The presence of Kolliphor^®^ P188 and phosphatidylcholine might helps in to formulate micelles surrounding of core comprising of loaded Dacarbazine (DTIC). Therefore, an adequate number of DTIC loaded overturned micelles might be seen near the core-shell region; the TEM image also confirmed the formation of such micelle’s formation (**Figures 2B**, **3**).

Reverse micelles are made in a unique ways, according to R M de Souza et al. (2020) ELBA (44), coarse-grained force can be used to check the self-assembly of 2-dipalmitoyl-sn-glycero-3-phosphocholine/1, 2-dihexaoyl-sn-glycero-3 phosphocholine(DPPC/DHPC)bicelles. The results of the atomic experiment suggest that disclike shape reverse micelles can be prepared with a higher coarse-grained force and a correct aggregation number while using a higher coarse-grained force.

However, some speculated models are postulated, which support drug distribution theory, i.e., (a)the drug enriched within the core-shell, (b). The partially aqueous soluble DTIC dissolved in ethyl alcohol, organic solvent, and subsequently added into warm dextrose 5% water to form reverse micelles. Therefore, it is essential to have good compatibility between hydrophobic surfactant phosphatidylcholine and hydrophilic surfactant called Kolliphor^®^ P188. To form a good lipid matrix, both hydrophobic and hydrophilic surfactants ratio optimization is required. Since phosphatidylcholine is strongly lipophilic, it can attempt to dissolve and encapsulate DTIC. The hydrophilic nature of Kolliphor^®^ P188, on the other hand, can shape fabric-like projection structures around the DTIC-SLNs, preventing micelles from dissolving in aqueous environments. The fraction of Kolliphor^®^ P188 that remains in the aqueous solvent during the SLNs preparation process, in principle, determines the encapsulation efficiency of hydrophobic DTIC. The entrapment efficacy (%) and particle size findings of DTIC-SLNs-10 and DTIC-SLNs-11 indicate that in the absence of phosphatidylcholine and Kolliphor^®^ P188, the solid lipid nanoparticles’ entrapment efficacy (%) decreases and particle size increases. This clearly indicates phosphatidylcholine and Kolliphor^®^ P188 can entrap micelles within the shell region, thus preventing the escape of micelles.

Moreover, 5.5, 7,5 and 10 mg for Phosphatidylcholine and as 1.1, 1.2 and 1.4% for Kolliphor^®^ P188 ratio had a great influence on entrapment efficacy (%), particle size (nm) and zeta potential (mV). In DTIC-SLNs-1, as the lower ratio of Phosphatidylcholine and Kolliphor^®^ P188 (5.5:1.0) resultant in moderately higher particle size (415 ± 10.89nm), low entrapment efficacy (51.56 ± 1.78%) and unstable zeta potential (-12.45 ± 2.78mV); this phenomenon might be due to the uneven coating of glycerol monooleate over SLN in the presence of lower Phosphatidylcholine and Kolliphor^®^ P188 ratio. However, in formulation DTIC-SLNs-9, where the higher ratio of Phosphatidylcholine and Kolliphor^®^ P188 (10:1.4) incorporated, resulted in higher entrapment efficacy (87.45 ± 4.78%), moderately low particle size (198 ± 4.35nm) and highly stable zeta potential (-25.56 ± 2.78mV); this might be due to the presence of steric repulsion between the particles and appropriate coating of glycerol monooleate while formulating SLN.

**Figure 4:**
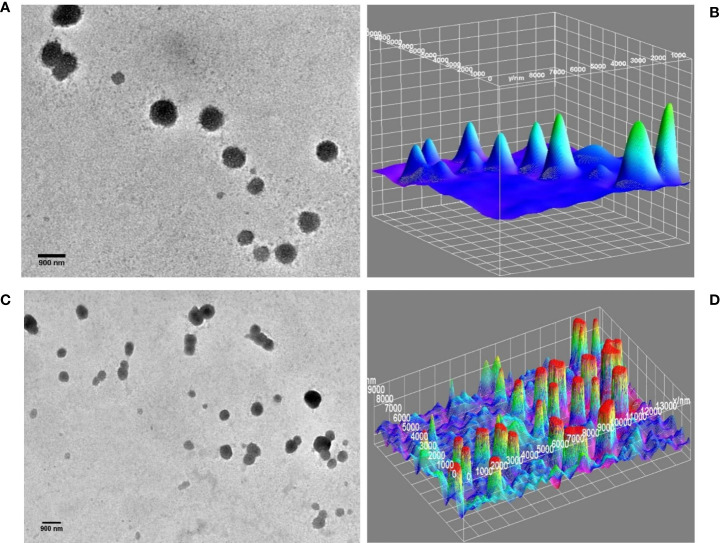
TEM image of DTIC-SLNs-10; without Kolliphor^®^ **(A)** with 3D surface morphology **(B)**. TEM image of DTIC-SLNs-11; without phosphatidylcholine **(C)** with 3D surface morphology **(D)**.

However, TEM images of DTIC-SLNs-10 and DTIC-SLNs-11 showing larger particle size with the absence of micellar formation. To understand the morphological behaviour of the DTIC-SLNs-10 and DTIC-SLNs-11, 3D surface images were plotted using ImageJ software (**Figures 4B, D**). It is evident from **Figures 4A, C** that SLNs without phosphatidylcholine and Kolliphor^®^ P188 may have agglomerated mushooms effects due to their larger particle size.

It was also understood that in the absence of one of both, phosphatidylcholine and Kolliphor^®^, SLNs could not produce micelles within and hance, entrapment efficacy (%) became limited. Moreover, the DTIC-SLNs-10 and DTIC-SLNs-11 corona and outer surface turns out to be fragile and leaky as compared to the DTIC-SLNs-9; which could result in less entrapment efficacy. It can be assumed that the number of micelles which can be migrated in the water phase would be much higher when phosphatidylcholine and Kolliphor^®^ were absent in formulation, which ultimately results in low encapsulation efficacy (%); This phenomenon can easily explain by observing higher particle size (821 ± 6.1nm &715 ± 7.36nm) and higher PDI (0.51 ± 0.023 & 0.27 ± 0.001) for DTIC-SLNs-10 & DTIC-SLNs-11.

Gold nanoparticles were coated with DTIC to learn more about drug localization inside prepared SLNs. According to Chaitali D. Dekiwadia et al. (2012), peptide-mediated cell penetration was studied using 13 nm gold nanoparticles, which are efficiently and selectively delivered into lysosomes with minimal cytotoxicity (45).

**Figure 5:**
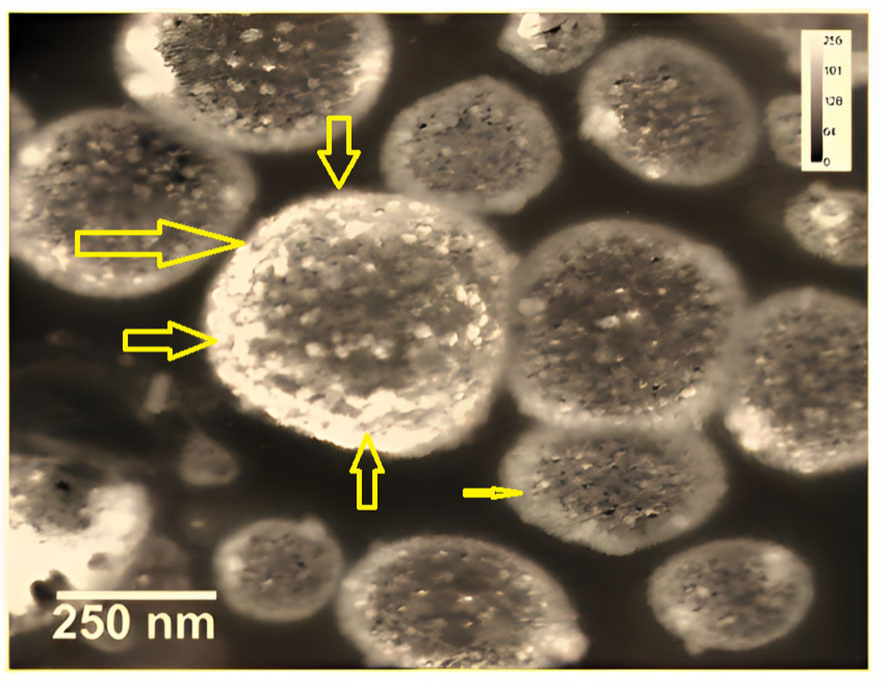
Gold nanoparticles coating with DTIC to check drug localization within SLNs.

DTIC loaded colloidal gold nanoparticles develop a process of selection zone and were clustered in the shell area, as seen in **Figure 5**. The evidence supports the localization hypothesis, implying that perhaps the formulation’s entrapment efficacy (%) was dependent on the correct use of phosphatidylcholine and Kolliphor^®^ in solid lipid nanoparticles. The gold nanoparticles collated DTIC composition also indicates that the nanoparticles’ laminated surface region facilitates steric hindrance.

### 3.3 The SLN’s permeation behaviour

**Table 3** is indicating the permeation parameters for all 11 formulations and the free drug. The presence of phosphatidylcholine enhances the lipophilicity of the formulations; therefore, prepared SLNs can able to penetrate more abruptly though the hydrophobic-lipophilic membrane as compared to free drug (DTIC). The relationship of cumulative amounts of permeated DTIC for 24 h Q24(µg/cm^3^) and Lag time(min) in almost all the formulation shows a significant relationship. The Q24, which is known as the number of micrograms passing though 0.1 mm of the membrane in cumulative time, increased significantly. The formulae, which are comprising of enhanced phosphatidylcholine and Kolliphor^®^ concertation, are showing higher Q24 value. The DTIC-SLNs-8 shows the highest membrane permeability (275 ± 5.67µg/cm^3^).

**Table 3:** The permeation parameters of different SLN formulas compared to the free drug (mean ± SD, n=3).

Formula number	Q_24_(µg/cm^3)^	Lag time(min)	Kp (cm/hr)
**DTIC-SLNs-1**	134± 3.78	70±2.89	0.0738±0.003
**DTIC-SLNs-2**	140±6.89	66±3.89	0.1102±0.002
**DTIC-SLNs-3**	141±7.27	68±4.89	0.0951±0.001
**DTIC-SLNs-4**	136±4.93	64±3.33	0.0823±0.002
**DTIC-SLNs-5**	216±10.89	54±4.89	0.2736±0.007
**DTIC-SLNs-6**	149±7.89	64±4.11	0.1562±0.011
**DTIC-SLNs-7**	140±5.78	65±3.21	0.0627±0.026
**DTIC-SLNs-8**	275±5.67	40±4.14	0.5246±0.039
**DTIC-SLNs-9**	169±4.78	70±4.36	0.1578±0.002
**DTIC-SLNs-10**	134±6.36	60±3.78	0.0938±0.001
**DTIC-SLNs-11**	117±4.77	69±3.22	0.0674±0.004
**Free drug**	128±6.82	84±4.89	0.0456±0.002

The DTIC-SLNs-8 shows the highest permeability coefficient, known as the slope of the straight-line portion of the curve separated by the drug concentration formerly applied (0.5246 ± 0.039 cm/h) with the shortest lag time 40 ± 4.14min. Lower particle size, lower PDI, higher surface area and lower aggrupation’s are some of the attributions for enhance permeation and shortest lag time of DTIC-SLNs-8. It is very indispensable to have shortage lag time for any topical formulation as topical formulations are meant to be removed form skin mechanically by patients. Therefore, it is a prerequisite to deliver payload on the target site within a limited time. In order to conduct *in vivo* studies, formulations with higher drug entrapment near the core shell area and a faster drug distribution profile should be chosen. As a consequence, smaller particle size and PDI formulation known as DTIC-SLNs-8 was proposed for integration into several gel metrics. DTIC-SLNs-9 also had a good drug encapsulation efficacy (%), but the formulation PDI was higher than DTIC-SLNs-8. Therefore, DTIC-SLNs-8 was considered as an optimised batch.

According to RP. Thatipamula et al. (2011), the ultrasonication technique was used to prepare domperidone-loaded solid lipid nanoparticles and nanostructured lipid carriers. Stability tests were performed on these formulations for 40 days. The developed nanoparticles were fairly stable, with no significant (P<0.05) changes in particle size, zeta potential, PDI, or entrapment efficiency. Within nanoparticles, perfect ratios of trimyristin as a solid lipid, cetyl recinoleate as a liquid lipid, and a mixture of soy phosphatidylcholine (99%) and tween 80 as a surfactant were observed, resulting in an improved stability profile (46).

**Figure 6:**
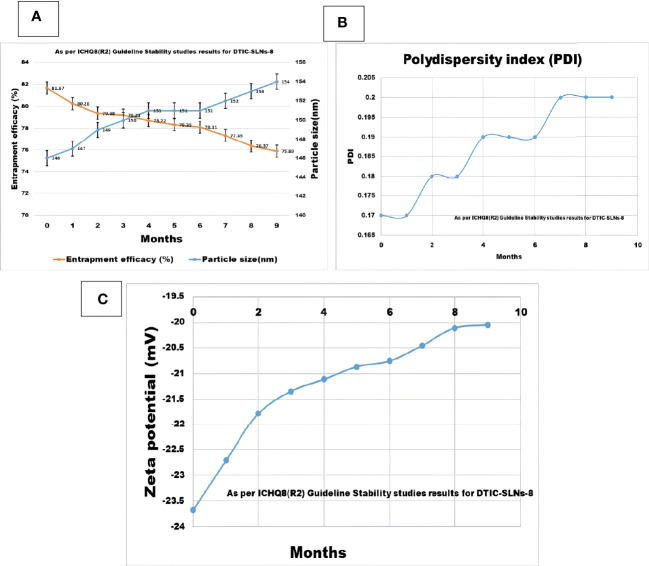
Nine month Stability studies results compilation for DTIC-SLNs-8; **(A)** Entrapment efficacy (%) and particle size (nm) after nine months **(B)** Polydispersity index (PI) after nine months **(C)** Zeta potential (mV) after nine months.

In the current research, the stability studies for DTIC-SLNs-8 were performed for 9 months. It was observed that zeta potential and polydispersity index not shown any big difference after 9-month stability studies, which indicates a good stability profile of the formulation. However, insignificant changes(p>0.05) of particle size increased from 146 ± 4.71nm to 154 ± 10.67nm, and a nearly 7.07% decrease of entrapment efficacy (%) was reported within nine months of stability studies (**Figure 6**). The higher particle size was reported due to the evolution of Ostwald ripening effects between nanoparticles within nine months. Since the PDI of the DTIC-SLNs-8 did not alter dramatically, variations in electrostatic charges within the nanoparticles’ outer surface may be another explanation for the larger particle size. Furthermore, during Ostwald ripening, smaller particles diffuse across the outer surface of nanoparticles, creating larger particles. These observations are in accordance with those of other research published in the literature.

### 3.4 SLN preparation on a variety of gel bases

As per Usama A Fahmy et al. (2020) Flibanserin lipid carriers incorporated into 0.6% w/v gellan gum comprising nasal in-situ gel improve the drug bioavailability and drug delivery profile in brain (47).As a result, it’s reasonable to believe that the required concentration of gellan gum will result in good mucoadhesive properties. In the current experiment design gellan gum (0.01% w/v), a linear anionic polysaccharide, chitosan (0.5% w/v), a polycationic linear polysaccharide, polysarcosine (2.5%w/v), a cationic polypeptide was all incorporated to prepared multiple DTIC-SLNs-8 gel formulations. According to the diffusion studies, DTIC-SLNs-8 formulations has the best Q24, the shortest lag period, and the highest Kp value in Gellan gum (0.01% w/v) (**Tables 4**, **5**).

**Table 4:** The permeation parameters of both DTIC-SLNs-8 and free DTIC in various gel bases [Gellan gum (0.01%w/v), Chitosan (0.5% w/v), Polysarcosine (2.5% w/v)] to illustrate the increase in drug permeation when loaded into SLNs and the impact of different gel bases on both the SLNs and the free drug permeation (means± SD, n=3).

Component of the gel formula	Q_24_(µg/cm^3)^	Lag time(min)	Kp (cm/hr)
DTIC-SLNs-8 suspended in Gellan gum (0.01%w/v)	257.12± 8.11	50±1.70	0.6261 ± 0.0121
Only DTIC suspended in Gellan gum (0.01%w/v)	127.34±3.10	100±1.21	0.0512 ± 0.0041
DTIC-SLNs-8 suspended in Chitosan (0.5% w/v)	230.26±8.09	60±3.10	0.382 ± 0.0218
Only DTIC suspended in Chitosan (0.5% w/v)	134.78±4.71	90±2.01	0.0828 ± 0.0023
DTIC-SLNs-8 suspended Polysarcosine (2.5% w/v)	139.81±6.27	74±7.04	0.2827 ±0.0036
Only DTIC suspended Polysarcosine (2.5% w/v)	163.25±9.14	78±2.78	0.1911 ±0.011

**Table 5:** DTIC-SLNs-8 combined with Gellan gum (0.01 % w/v), Chitosan (0.5 % w/v), and Polysarcosine (2.5 % w/v) permeation parameters (means SD, n=3).

Component	Q_24_ (µg/cm^3)^	Lag time (min)	Kp (cm/hr)
DTIC-SLNs-8 mixed with Gellan gum (0.01%w/v)	257.12± 8.11	50±1.70	0.6261 ± 0.0121
DTIC-SLNs-8 mixed with Chitosan (0.5% w/v)	230.26±8.09	60±3.10	0.382 ± 0.0218
DTIC-SLNs-8 mixed with Polysarcosine (2.5% w/v)	139.81±6.27	74±7.04	0.2827 ±0.0036

### 3.5 Effect of DTIC-SLNs-8 and DTIC on DMBA-induced tumor growth, incidence, multiplicity and volume

**Figure 7:**
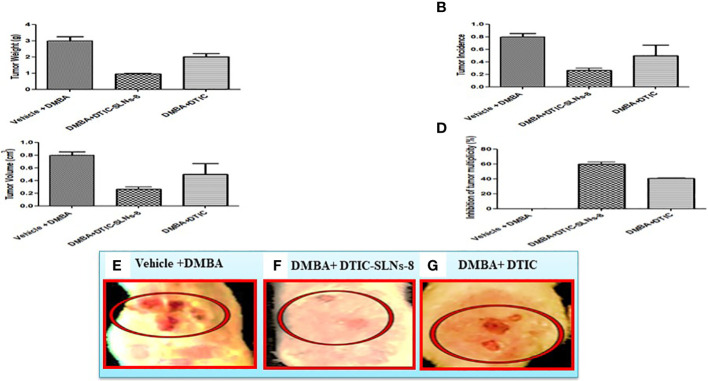
DTIC-SLNs-8 inhibits tDMBA-induced skin tumor occurrence, multiplicity, volume and weight. Representative bar graph are shown the effect of DTIC-SLNs-8 on DMBA-induced skin tumorigenesis; **(A)** the change of tumor weight of rats in every two weeks until the end of experiment period i.e. 18 weeks, **(B)** percentage of tumor incidence of rats thoughout the duration of experiment, **(C)** percent inhibition of tumor multiplicity with respect to DMBA alone treated group, **(D)** average tumor volume of all experimental groups, **(E)** Vehicle with DMBA tumor image **(F)** DMBA +DTIC-SLNs-8 tumor image **(G)** DMBA + DTIC tumor image. Control, acetone (vehicle) treated group; DMBA, DMBA/TPA treated group; DMBA+DTIC-SLNs-8, DMBA/TPA-treated rats with DTIC-SLNs-8 with Gellan gum (0.01%w/v); DMBA+DTIC, DMBA/TPA-treated rats with DTIC with Gellan gum (0.01%w/v). Each data point is represented as mean ± SD of 10 rats in each group. Statistical analyses were performed with Student’s t-test. *p < 0.01 compared with DMBA alone treated group. **P ≤ 0.01. represent significant different than disease control group.

The tumor weight (**Figure 7A**), tumor incidences were significantly reduced in DMBA group treated with DTIC-SLNs-8 with Gellan gum (0.01%w/v) (**Figure 7B**). In addition, DTIC-SLNs-8 treatment showed significant inhibition of tumor volume (**Figure 7C**) as well as inhibition of tumor multiplicity (number and size of tumors/rat) was markedly increased by DTIC-SLNs-8 treatment as compared to DMBA treated group (**Figure 7D**). Represent the invasion and metastatic condition of tumours in skin (**Figure 7E**). Showing that how DMBA +DTIC SLNs-8 inhibiting the invasion and metastatic condition of tumours in skin (**Figure 7F**). As we expected, and comparing to DMBA treated group, suggesting that DTIC-SLNs-8 inhibits DMBA-induced tumor growth.

### 3.6 Histological assessments

#### 3.6.1 Effects of solid lipid nano formulation on skin cancer growth and angiogenesis in rats

According to Pramod Kumar et al. (2019), after the gestation of Methylthioadenosine solid lipid nanoparticles in the corpus callosum of mice, solid lipid nanoparticles in the remyelination of neurons showed potential accumulation (48). As a result, proper SLN preparation could significantly improve drug accumulation in the targeted site.

**Figure 8:**
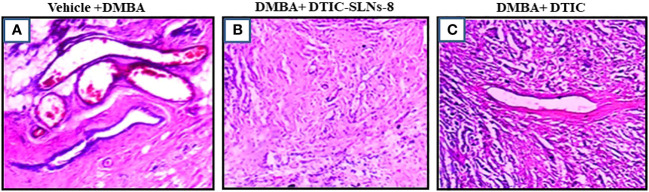
Histology (hematoxylin-eosin (H&E) staining) of the skin of hairless rats after treatment with DMBA+ DTIC-SLNs-8 and DMBA+ DTIC (0.01%w/v). **(A)** (0.01%w/v) Group; **(B)** DMBA+ DTIC-SLNs-8 Group; **(C)** DMBA+ DTIC Group. Each specimen was subjected to H&E staining and photographed at a magnification of 400x. Scale bar = 100 µm. **(A)** Arrow represents Squamous cell carcinomas with large nuclei and high mitotic figure & inflammation: H&E x400. **(B)** Squamous cell carcinomas with very minure nuclei and minure mitotic figure. **(C)** Squamous cell carcinomas with moderate nuclei and moderate mitotic figure H&E x400.

Prior to histopathological findings, the developed melanoma cells were extracted. The targeted invasion of the cancerous cell on the skin can be calculated using the following formula: Width × Height × Proportion. The DMBA mediated positive control showed a tumor volume of 21.3 mm^3^, and rats treated with Dacarbazine Shell-enriched Solid Lipid Nanoparticles (SLN) (DTIC-SLNs) treated cancerous tissue had a tumor volume of 8.5 mm^3^. However, free Dacarbazine (DTIC)treated induced cancerous tissue has 18.3 mm^3^. This indicates that DTIC-SLNs treated rats had double cancer recovery compared to free DTIC-treated rats. From the histopathological findings in comparison to the DMBA mediated positive control indicates visible mitotic nuclei, haemorrhage, hyperkeratosis, and inflammatory reactions (**Figures 8A, B**). The histopathological analysis by H&E staining of the tumors is summarized in **Table 6** and **Figure 8**, The presence of Dacarbazine in melanoma therapy activates ligands for the anchoring the immunoreceptor NKG2D to generate IFN- cells, which ultimately kills tumour cells. IFN- upregulates the development of large complex histocompatibility class I on tumour cells, enabling better detection of cytotoxic CD8+ T lymphocytes (CTLs). From the histological outcomes it was also evident that, Dacarbazine stable-projectile Solid Lipid Nanoparticles (SLN) (DTIC-SLNs) topical application, enhances permeation capabilities of DTIC, which can be justified by **Figure 8B**, where limited inflammatory reactions and infinitesimal haemorrhagic area were witnessed as compared to the only DTIC treated (**Figure 8C**) and positive control slide (**Figure 8A**). The histopathological findings support *in-vitro* diffusion studies, where it is clearly apparent that prepared Dacarbazine Shell-enriched Solid Lipid Nanoparticles (SLN) (DTIC-SLNs) can deliver chemotherapeutic drug effectively. The microscopical image of DTIC-SLNs treated tissue revealed that inflammation reaction was minimized. These inflammations were recorded due to the presence of over-expressed growth factors on the tissue surface and due to angiogenesis. However, using tumour markers to qualify and consolidate this research’s results is warranted. these data indicated that DTIC-SLNs-8 suppressed DMBA induced skin cancer growth, invasion, angiogenesis in rats.

**Table 6:** Characteristics of DMBA tumours generated in Rats.

Tumour Characteristics	DMBA Control	DMBA+ DTIC-SLNs-8	DMBA+ DTIC
Tumour infiltration	+++	--	+
Angiogenesis	+++	--	+
Tumour cells (Giant)	High	Less	Scanty
Mitotic condition	+++	--	--
Inflammation	+++	--	--
Nuclei figures	High	Less	Less nuclear size variation

Representation: - + : Moderate to poor; ++ : Moderate; +++: High; - - : Very Low.

### 3.7 Immunohistochemistry

**Figure 9:**
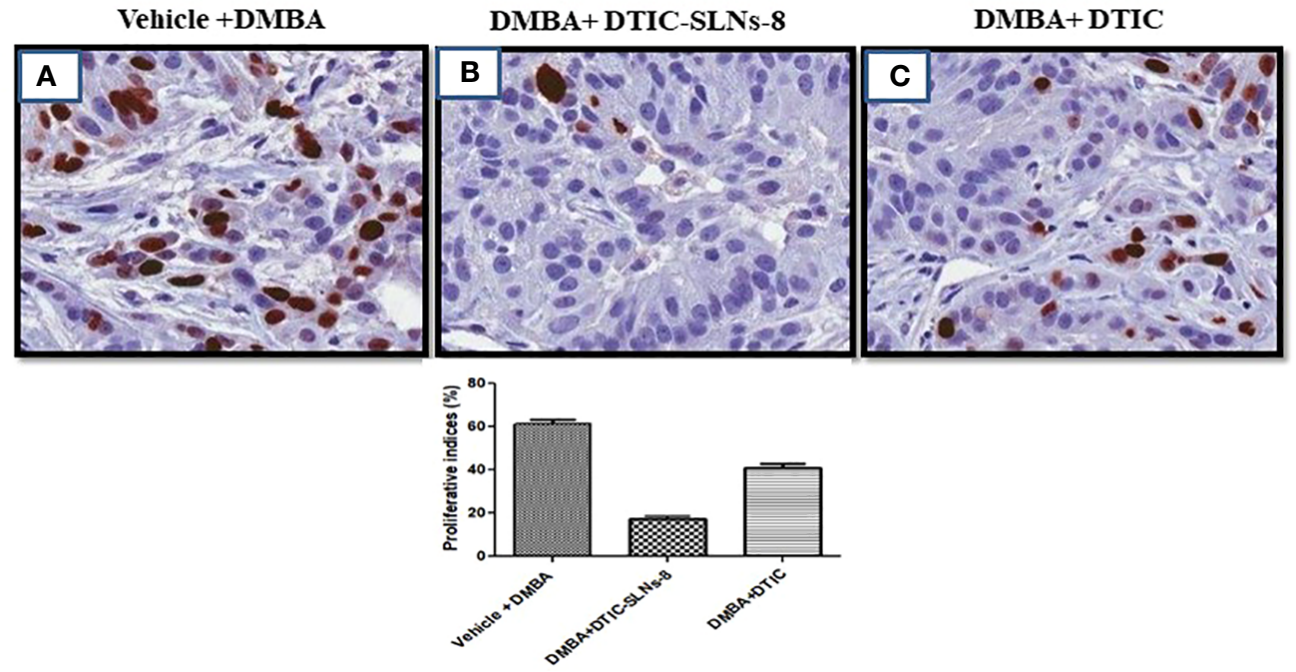
**(A)** represents overexpression of Ki-67 in DMBA treated group. **(B)** indicating DMBA+DTIC-SLNs-8 decreases proliferative cells in the tumor section in DMBA-induced skin tumors. **(C)** representing the moderate downregulation of Ki-67 expression. Representative images micrographs of Ki-67 staining (nuclei stained with brown color) in the tumor section of experimental groups are shown. Bar graph is shown the percentage of proliferative cells by counting Ki-67 positive cells in 15 randomly selected microscopic (40× objective) fields in each group were calculated by the total number of cells divided by the number of Ki-67 positive cells. vehicle Control treated group; DMBA treated group; DMBA+ DTIC-SLNs-8 with Gellan gum (0.01%w/v); DMBA+DTIC with Gellan gum (0.01%w/v) Statistical analyses were performed with Student’s t-test *p < 0.01 compared with DMBA alone treated group.

Nuclear protein Ki67 has a close relationship with the cell cycle. It has been used to classify invasive skin cancer patients into prognostic groups with favourable and worse outcomes since it is a measure of cell proliferation. Its relationship to variations in gene expression has not yet been completely understood. In this work, slices taken from 21 invasive skin tumours that had been formalin-fixed and paraffin-embedded underwent Ki67 immunohistochemistry using the MIB-1 antibody. No immunostaining resulted in a score of zero, low immunopositivity (less than 10%), or strong immunoreactivity (more than 10%), respectively. The ki67 expression shown very higher(**Figure 9A**), tumor ki-67 expression were significantly reduced in DMBA group treated with DTIC-SLNs-8 with Gellan gum (0.01%w/v) (**Figure 9B**). In addition, DTIC-SLNs-8 treatment showed significant downregulation of Ki-67 expression (**Figure 9C**) these findings suggesting that DTIC-SLNs-8 can inhibits DMBA-induced skin tumor growth in wistar rats.

## 4 Conclusion

Dacarbazine (DTIC) was successfully encapsulated into shell-enriched solid lipid nanoparticles. Absence of phosphatidylcholine and Kolliphor^®^ P188 within the composition of solid lipid nanoparticles could decreases entrapment efficacy (%) and increases particle size. Kolliphor^®^ P188 offers additional steric and electrostatic stabilization within formulae with improve negative zeta potential. The formula, which are comprising of enhanced phosphatidylcholine and Kolliphor^®^ concertation, are showing higher Q24 value. DTIC-capped gold nanoparticles have the potential to be a promising drug delivery system for melanoma treatment due to the unique properties of gold nanoparticles and the ability to encapsulate DTIC within the SLN matrix. Based on characterization studies, DTIC-SLNs-8 formulation was found to be the best in terms physical stability. The DTIC-SLNs-1 to DTIC-SLNs-9 findings demonstrated that the inclusion of an accurate proportion of phosphatidylcholine and Kolliphor^®^ within the formulation could improve the formulation’s diffusion and release pattern as compared to the free drug alone. In contrast to the other polymer matrix, gellan gum (0.01% w/v), a linear anionic polysaccharide, was found to be the better diffusion matrix for Dacarbazine. As a result, Dacarbazine Shell-enriched Solid Lipid Nanoparticles may be filled with a gel matrix containing Gellan gum (0.01% w/v). According to stability data, optimized Dacarbazine Shell-enriched Solid Lipid Nanoparticles produces a significant stability profile over a nine-month period. Histopathological tests specifically indicate that an optimised formulation could be very effective in the treatment of melanoma, as the skin shows no hyperkeratosis and little evidence of inflammation after application over skin and showed normal epidermis & dermal integrity. However, a positive control mediated DMBA shows visible haemorrhage, hyperkeratosis, and inflammatory reactions. As a consequence, stable-projectile solid lipid nanoparticles (SLNs) could be the ideal topical delivery system for Dacarbazine (DTIC) treatment of melanoma. For poorly water-soluble chemotherapeutic drugs, this strategy could provide a promising formulation platform for designing drug delivery systems with improved drug retention over skin. A xenograft experiment using nanoparticles would be useful for studying *in vivo* skin cancer cell growth and behavior in forthcoming research.

## Data availability statement

The raw data supporting the conclusions of this article will be made available by the authors, without undue reservation.

## Ethics statement

The experiments were approved by the Institutional Animal Ethics Committee (1410/c/11/CPCSEA) Deshpande Laboratories, Bhopal, India.

## Author contributions

SB wrote, drafted, and designed the manuscript. SS validated the data. Both authors have consent for publication. SB submitted the final draft to the journal. All authors contributed to the article and approved the submitted version.
